# Relationships between body composition and pulmonary function in a community-dwelling population in Japan

**DOI:** 10.1371/journal.pone.0242308

**Published:** 2020-11-17

**Authors:** Ryosuke Kawabata, Yuki Soma, Yutaro Kudo, Junichi Yokoyama, Hiroyasu Shimizu, Arata Akaike, Daisuke Suzuki, Yoshihisa Katsuragi, Manabu Totsuka, Shigeyuki Nakaji

**Affiliations:** 1 Department of Social Medicine, Hirosaki University Graduate School of Medicine, Hirosaki, Aomori, Japan; 2 Department of General Science Education, National Institute of Technology, Hachinohe College, Hachinohe, Aomori, Japan; 3 Department of Life Science, Morioka Junior College, Iwate Prefectural University, Takizawa, Iwate, Japan; 4 Department of Physical Education, Nippon Sport Science University, Setagaya-ku, Tokyo, Japan; 5 Department of Active Life Promotion Sciences, Hirosaki University Graduate School of Medicine, Hirosaki, Aomori, Japan; 6 Health Care Food Research Laboratories, Kao Corporation, Sumida-ku, Tokyo, Japan; 7 Department of Education, Hirosaki University, Hirosaki, Aomori, Japan; San Raffaele Roma Open University, ITALY

## Abstract

Pulmonary diseases, including chronic obstructive pulmonary disease (COPD), are major chronic diseases that result in decreased pulmonary function. Relationships between body composition and pulmonary function have been reported. However, few epidemiological studies have used the visceral fat area (VFA) to measure body composition. This study aimed to examine the relationship between body composition and pulmonary function. A cross-sectional study was conducted between 2015 and 2016, using data obtained from 1,287 residents aged between 19 and 91 years living in the Iwaki area of Hirosaki City, a rural region in Aomori Prefecture, Japan. Pulmonary function was evaluated using the forced vital capacity (FVC) as a percentage of the predicted value (predicted FVC%) and the ratio of forced expiratory volume in one second (FEV_1_) to FVC. The measurements for evaluating body composition included the body fat percentage (BFP) of the whole body and trunk, skeletal muscle index (SMI), body mass index (BMI), VFA, waist circumference (WC) at the navel level, and waist-to-hip ratio (WHR). To adjust for potential confounders, Spearman’s partial correlation analysis was used to examine the relationship between the measurements of body composition and pulmonary function. There were significant correlations between the predicted FVC% and the following parameters: BFP (whole body and trunk) in younger males; SMI in older males; WC, VFA, BMI, and SMI in younger females; and BFP (whole body and trunk) and VFA in older females. Contrastingly, WC and VFA in younger males and WC in younger females were correlated with the FEV_1_/FVC ratio. VFA was correlated with the FEV_1_/FVC ratio in younger males and predicted FVC% in older females. These findings suggest that visceral fat accumulation may increase the development of obstructive pulmonary disease in young males and accelerate the decline of pulmonary function (predicted FVC%) in older females.

## Introduction

Smoking status [1], aging [2], decreased muscle mass [3], and obesity [4] impair pulmonary function to varying degrees and may induce severe respiratory diseases, including chronic obstructive pulmonary disease (COPD) [5]. Intra-abdominal fat accumulation may impede diaphragm descent during inspiration, affecting several spirometry variables [6]. Moreover, fat deposition on the chest wall may impede rib cage expansion and excursion [7]. In addition, metabolic syndrome contributes to obstructive pulmonary disease and is influenced by glucose tolerance and inflammatory cytokines [8]. Therefore, the visceral fat area (VFA) is likely related to pulmonary function. Recently, computed tomography (CT) [9], magnetic resonance imaging [10], and dual-energy X-ray absorptiometry [11] have been demonstrated as effective modalities for the relatively accurate measurement of total body fat and visceral fat. However, these modalities have limited applications in large-scale and general population surveys because of radiation exposure-related problems, technical complexity, and high cost.

Increasing attention has been given to impedance methods that are unaffected by the aforementioned problems. In particular, the eight-polar bioelectrical impedance analysis [12] allows accurate measurement of each site. However, considering the increasingly important role of visceral fat in metabolic syndrome, specialized techniques for measuring intra-abdominal fat are required. Consequently, a method for measuring visceral fat was developed, which used both impedance methods in the anterior and posterior axial directions of the trunk [13] and dual bioelectrical impedance analysis [14]. The results from both methods were highly correlated with CT measurements. However, there is a need for further study in the healthy general population and young people to identify the relationship between body fat (including intra-abdominal fat) and pulmonary function, with an emphasis on prophylaxis.

The improved measurement accuracy of the impedance method, which measures visceral and subcutaneous fat separately, has allowed its use in body fat measurements for epidemiological studies.

The features of this study are as follows:

1. A relatively large, healthy population was studied.
2. Visceral fat was measured using the impedance method.
3. Various confounding factors that affect pulmonary function and body composition (including body fat) were measured.
4. Homeostasis model assessment of insulin resistance (HOMA-IR) and interleukin-6 (IL-6) levels were determined to elucidate the mechanism underlying the prevention and improvement of obstructive pulmonary diseases.

To our knowledge, there has been no report on the association between pulmonary function and body composition (including VFA) in the healthy general population. The mechanisms underlying prevention and improvement of pulmonary function and body composition remain unclear. Elucidating the association of body composition measurements, specifically the VFA and obesity criteria, with pulmonary function and determining the underlying mechanism may provide basic data for the prevention and improvement of obstructive pulmonary diseases. Therefore, we aimed to examine the relationship between body composition and pulmonary function.

## Materials and methods

### Participants

This study enrolled residents aged between 19 and 91 years living in the Iwaki area of Hirosaki City, a rural region in Aomori Prefecture, Japan, to the Iwaki Health Promotion Project in 2015 and 2016. When participants were included both in 2015 and 2016, their 2015 data was adopted. We excluded participants who reported a previous diagnosis of heart disease, stroke, or cancer (n = 153) and those who did not fully complete the pulmonary function/body composition measurements or the questionnaire, which included medical history and lifestyle behavioral questions (n = 10). Finally, 1,287 participants (498 males and 789 females) were included for analysis. For further analysis of the association between VFA and the ratio of forced expiratory volume in one second (FEV_1_) to forced vital capacity (FVC) in the younger male group, 383 participants in the younger male group were selected from the 1287 participants. Among these 383 participants (aged 19–64 years), 9 with diabetes (fasting blood glucose ≥ 126 mg/dL [15]) and 3 with missing data were excluded. Consequently, 371 people were included in the analysis. All participants provided informed written consent; further, this study was conducted in accordance with the Declaration of Helsinki. Ethical approval was provided by the Ethics Committee of the Hirosaki University Graduate School of Medicine (approval numbers: 2014–377, 2016–028).

### Survey items

#### Questionnaire investigation

Each participant completed a self-administered questionnaire regarding sex, age, and smoking status (current smoker, ex-smoker, non-smoker).

#### Body weight, body fat percentage, and skeletal muscle mass

A body composition analyzer (MC-190; Tanita Corp., Tokyo, Japan) [12], using the multi-frequency bioelectrical impedance method, was used to measure body mass (kg), body fat percentage (%), and skeletal muscle mass (kg). The body mass index (BMI) was calculated by inputting the measured height to this body composition analyzer. We used BMI ≥ 25 kg/m^2^ as the obesity criterion [16]. The skeletal muscle mass was evaluated using the skeletal muscle index (SMI), calculated as the appendicular muscle mass divided by the height squared [17].

#### VFA

The visceral fat-measuring apparatus (EW-FA90; Panasonic, Osaka, Japan), which used the bioelectrical impedance analysis method [13], was used for waist and VFA measurements. We used a VFA ≥ 100 cm^2^ as the visceral fat obesity criterion [16]. The waist circumference (WC) was simultaneously measured at the navel level using this device. We used ≥ 85 cm in males and ≥ 90 cm in females as the obesity criteria [16].

#### Waist-to-hip ratio

The waist-to-hip ratio (WHR) was calculated as WC divided by hip circumference. We used ≥ 0.90 in males and ≥ 0.85 in females as the obesity criteria of WHR [18].

#### Pulmonary function

FVC and the FEV_1_/FVC ratio measurements were obtained using a multi-functional spirometer (HI-801; CHEST MI, Inc., Tokyo, Japan) [19]. Pulmonary function was measured following the guidelines of the Japanese Respiratory Society, which are in accordance with those of the American Thoracic Society/European Respiraroty Society [20]. Predicted FVC% was calculated as FVC divided by the predicted FVC estimated using the LMS method [21] as follows:

predictedFVC=exp(−8.8877+2.1494×ln(h)−0.1891ln(a)+m‐s)

where a = age in years; h = height in cm; ln() = natural log transformation; m-s = M-spline by age.

#### Blood analysis data

*a) HOMA-IR*. HOMA-IR was performed by homeostasis model assessment based on fasting blood glucose and insulin levels, with levels ≥ 2.5 being considered abnormal [22].

*b) IL-6*. Serum IL-6 concentration was measured by chemiluminescent enzyme immunoassay using a commercially available kit (LSI Medience, Tokyo, Japan); values > 4 pg/ml were considered abnormal.

### Statistical analysis

All participants were categorized according to sex and age (19–64 years [the younger group] and ≥ 65 years [the older group]). Descriptive statistics are presented as median, 25^th^ and 75^th^ percentile, or percentage. The Mann–Whitney U‐test was applied to compare measurement variables between the obesity and age groups. The Kruskal-Wallis test was applied to compare measurement variables between the sum of obesity criteria (0, 1, and 2–4) and pulmonary function. Within-group differences were evaluated with post hoc Bonferroni adjustment. Spearman’s partial correlation analysis was used to examine the relationship between body composition measurements and pulmonary function. This analysis was performed with adjustment for smoking status, age (only for the FEV_1_/FVC ratio), and SMI (except when analyzing the SMI). Because the predicted FVC calculated from the LMS method uses age as a predictor, age was not adjusted for in the analysis of the predicted FVC% value. Smoking status was categorized as current-smoker, ex-smoker, and non-smoker, and was included as a dummy variable. Between-group differences in the smoking status were examined using the chi-square test.

Additionally, Spearman’s partial correlation analysis was used to examine the relationships among the measurements of HOMA-IR, IL-6, and the FEV_1_/FVC ratio with adjustment for smoking status, age, and SMI. These variables were converted into dummy variables and entered in the analysis as adjustment variables. The Mann-Whitney U test was applied to compare the FEV_1_/FVC ratio between the normal and abnormal values of HOMA-IR and IL-6 and to compare HOMA-IR and IL-6 between the normal and abnormal values of VFA. In addition, Spearman's rank correlation analysis was used to examine the relationship between VFA and IL-6. Statistical significance was set at p < 0.05. IBM SPSS Statistics for Windows, version 25.0 (IBM Corp., Armonk, N.Y., USA) was used for analysis.

## Results

### Characteristics of participants

Table 1 shows the characteristics of the male participants. Age (p < 0.001), WHR (p < 0.001), body fat percent (p = 0.022), and body fat percent on the trunk (p = 0.022) were significantly higher in the older group than in the younger group; the opposite was the case with their height (p < 0.001), weight (p < 0.001), predicted FVC% values (p = 0.001), FEV_1_/FVC ratio (p < 0.001), and SMI (p < 0.001). Furthermore, there was a significantly higher frequency of non-smokers in the older group.

**Table 1:** Characteristics of the male study participants.

Variables	18–64 years		≥ 65 years		P-value
	(n = 383)		(n = 115)		
	Median	(25%–75%)	Median	(25%–75%)	
**Age (years)**	44.0	(35.0–54.0)	70.0	(66.0–75.0)	< 0.001
**Height (cm)**	171.0	(166.8–175.4)	163.6	(158.9–166.5)	< 0.001
**Weight (kg)**	67.9	(62.0–74.7)	61.6	(56.5–67.2)	< 0.001
**Waist circumference (cm)**	86.3	(81.8–91.4)	87.7	(82.3–91.9)	0.304
**Visceral fat area (cm**^**2**^**)**	97.0	(70.0–129.0)	104.0	(77.0–144.0)	0.123
**Waist-to-hip ratio**	0.860	(0.822–0.901)	0.892	(0.842–0.930)	< 0.001
**Body Mass Index**	23.3	(21.5–25.5)	23.0	(21.7–25.1)	0.498
**Body fat percentage (%)**	19.2	(15.4–22.5)	20.1	(17.0–24.6)	0.022
**Body fat percentage of the trunk (%)**	19.9	(15.3–23.7)	20.6	(17.2–26.3)	0.022
**Predicted FVC%**	111.4	(101.6–120.7)	103.5	(90.6–117.2)	0.001
**FEV**_**1**_**/FVC ratio (%)**	81.8	(77.4–85.6)	78.1	(73.0–83.7)	< 0.001
**Skeletal Muscle Index**	8.32	(7.69–8.93)	7.75	(7.09–8.17)	< 0.001
**Non-smoker**	128 (33.4)		62 (53.9)		< 0.001
**Current-smoker**	144 (37.6)		16 (13.9)		
**Ex-smoker**	111 (39.0)		37 (32.2)		

Values are the median and the 25^th^ and 75^th^ percentiles. Mann–Whitney U‐test or chi-square test was used for comparisons between the younger and older groups.

Abbreviations: Predicted FVC%, the FVC as a percentage of the predicted value.

Table 2 shows the characteristics of the female participants. Age (p < 0.001), WC (p < 0.001), VFA (p < 0.001), WHR (p < 0.001), body fat percent (p < 0.001), and body fat percent on the trunk (p < 0.001) were significantly higher in the older group than in the younger group; the opposite was the case with their height (p < 0.001), weight (p = 0.003), FEV_1_/FVC ratio (p < 0.001), and SMI (p < 0.001). Similar to the male participants, there was a significantly higher frequency of non-smokers in the older group.

**Table 2:** Characteristics of the female study participants.

Variables	18–64 years		≥ 65 years		P-value
	(n = 562)		(n = 227)		
	Median	(25%–75%)	Median	(25%–75%)	
**Age (years)**	47.0	(36.0–57.0)	70.0	(67.0–75.0)	< 0.001
**Height (cm)**	157.8	(154.4–161.2)	150.0	(146.8–153.6)	< 0.001
**Weight (kg)**	53.3	(48.3–59.6)	51.7	(46.9–57.7)	0.003
**Waist circumference (cm)**	80.6	(75.1–87.0)	85.2	(79.8–91.7)	< 0.001
**Visceral fat area (cm**^**2**^**)**	57.0	(37.0–81.0)	74.0	(54.0–96.0)	< 0.001
**Waist-to-hip ratio**	0.784	(0.743–0.830)	0.831	(0.798–0.869)	< 0.001
**Body Mass Index**	21.3	(19.4–23.7)	22.8	(21.1–25.0)	< 0.001
**Body fat percentage (%)**	28.4	(23.8–33.2)	31.4	(26.8–36.1)	< 0.001
**Body fat percentage of the trunk (%)**	26.9	(21.3–32.7)	30.6	(25.3–36.3)	< 0.001
**Predicted FVC%**	110.5	(100.1–121.7)	107.5	(97.6–120.4)	0.067
**FEV**_**1**_**/FVC ratio (%)**	83.1	(78.9–87.6)	79.7	(76.0–83.6)	< 0.001
**Skeletal Muscle Index**	6.44	(6.08–6.88)	6.28	(5.88–6.68)	< 0.001
**Non-smoker**	419 (74.6)		209 (92.1)		< 0.001
**Current-smoker**	72 (12.8)		7 (3.1)		
**Ex-smoker**	71 (12.6)		11 (4.8)		

Values are the median and the 25^th^ and 75^th^ percentiles. Mann–Whitney U‐test or chi-square test was used for comparisons between the younger and older female groups.

Abbreviations: Predicted FVC%, the FVC as a percentage of the predicted value.

### Relationship between the presence of obesity criteria outlier and pulmonary function

Tables 3–6 show the relationship between the presence of obesity criteria outlier and pulmonary function. In the younger groups of both sexes, there was a significantly stronger relationship between the FEV_1_/FVC ratio and VFA in the non-obese groups than in the obesity groups (males, p = 0.002; females, p = 0.044). Further, more younger males in the non-obese group met the 0 obesity criterion than the 2 obesity criterion (p < 0.001). There was no relationship between body composition and pulmonary function in the older male groups. In the older female groups, the relationships of the predicted FVC% with WC (p = 0.003), VFA (p = 0.002), and BMI (p = 0.023) were significantly higher in the non-obese group than in the obesity group.

**Table 3:** Relationship between the presence of obesity criteria outlier and pulmonary function in males aged between 19 and 64 years.

Variables		N	Predicted FVC%		P-value	FEV_1_/FVC ratio		P-value
			Median	(25%–75%)		Median	(25%–75%)	
**Waist circumference (cm)**	< 85 cm	254	112.0	(101.6–121.2)	0.216	82.0	(77.7–86.1)	0.063
	≥ 85 cm	129	110.4	(101.1–119.3)		81.2	(76.6–84.6)	
**Visceral fat area (cm**^**2**^**)**	< 100 cm^2^	200	111.9	(101.6–121.0)	0.663	82.7	(78.4–86.4)	0.002
	≥ 100 cm^2^	183	110.7	(101.1–119.7)		81.0	(76.4–84.5)	
**Waist-to-hip ratio**	< 0.9	283	111.3	(101.6–121.0)	0.697	82.0	(77.6–85.9)	0.154
	≥ 0.9	100	111.5	(101.2–119.5)		81.2	(76.7–84.8)	
**Body Mass Index (kg/cm**^**2**^**)**	< 25 kg/cm^2^	271	111.4	(101.5–121.1)	0.552	82.0	(77.4–85.9)	0.258
	≥ 25 kg/cm^2^	112	111.2	(101.6–119.6)		81.2	(76.7–84.8)	
**Sum of obesity criteria**	0	141	111.0	(100.0–119.9)	0.375	83.3	(78.9–87.3)	<0.001
	1	46	113.6	(102.3–127.9)		81.9	(76.4–84.4)	
	2–4	196	111.4	(101.6–119.7)		80.9	(76.4–84.5)	

Values are the median and the 25^th^ and 75^th^ percentiles. Mann–Whitney U‐test or Kruskal-Wallis test was used for comparisons between the applicable and not applicable groups.

Abbreviations: Predicted FVC%, the FVC as a percentage of the predicted value.

**Table 4:** Relationship between the presence of obesity criteria outlier and pulmonary function in females aged between 19 and 64 years.

		N	Predicted FVC%		P-value	FEV_1_/FVC ratio		P-value
			Median	(25%–75%)		Median	(25%–75%)	
**Waist circumference (cm)**	< 85 cm	535	110.5	(100.2–121.8)	0.310	83.1	(79.0–87.6)	0.732
	≥ 85 cm	27	106.2	(97.3–118.1)		82.9	(77.3–86.0)	
**Visceral fat area (cm**^**2**^**)**	< 100 cm^2^	499	110.1	(99.8–121.7)	0.329	83.2	(79.0–87.9)	0.044
	≥ 100 cm^2^	63	113.7	(104.0–123.2)		81.5	(77.2–85.4)	
**Waist-to-hip ratio**	< 0.9	473	110.8	(99.8–122.9)	0.571	83.2	(78.7–87.9)	0.341
	≥ 0.9	89	109.0	(102.1–118.8)		82.5	(79.4–86.2)	
**Body Mass Index**	< 25 kg/cm^2^	469	110.5	(99.9–121.9)	0.975	83.0	(78.6–87.8)	0.792
	≥ 25 kg/cm^2^	93	110.5	(101.4–120.1)		83.3	(80.3–87.2)	
**Sum of obesity criteria**	0	419	110.0	(99.3–121.8)	0.548	83.2	(78.8–88.4)	0.057
	1	70	111.6	(104.6–121.2)		81.3	(78.8–84.1)	
	2–4	73	110.6	(102.5–120.3)		82.9	(80.0–87.3)	

Values are the median and the 25^th^ and 75^th^ percentiles. Mann–Whitney U‐test or Kruskal-Wallis tests was used for comparisons between the applicable and not applicable groups.

Abbreviations: Predicted FVC%, the FVC as a percentage of the predicted value.

**Table 5:** Relationship between the presence of obesity criteria outlier and pulmonary function in males aged ≥ 65 years.

		N	Predicted FVC%		P—value	FEV_1_ / FVC ratio		P-value
			Median	(25%–75%)		Median	(25%–75%)	
**Waist circumference (cm)**	< 85 cm	68	103.1	(89.8–117.1)	0.531	76.9	(69.9–84.0)	0.289
	≥ 85 cm	47	103.5	(91.6–121.0)		79.1	(75.8–83.4)	
**Visceral fat area (cm**^**2**^**)**	< 100 cm^2^	50	102.8	(89.7–119.7)	0.620	77.6	(71.6–84.6)	0.763
	≥ 100 cm^2^	65	103.5	(91.9–116.1)		78.6	(73.7–83.5)	
**Waist-to-hip ratio**	< 0.9	66	105.5	(89.6–118.1)	0.769	78.3	(71.9–83.8)	0.841
	≥ 0.9	49	103.2	(92.5–115.0)		77.9	(73.7–83.6)	
**Body Mass Index**	< 25 kg/cm^2^	85	104.2	(89.7–117.6)	0.430	76.9	(71.5–83.2)	0.063
	≥ 25 kg/cm^2^	30	103.4	(94.3–116.5)		79.5	(76.2–84.7)	
**Sum of obesity criteria**	0	35	100.1	(88.0–117.2)	0.960	78.1	(72.6–84.9)	0.475
	1	12	113.6	(97.4–133.3)		76.9	(67.8–81.0)	
	2–4	68	103.4	(92.4–116.3)		78.7	(73.6–83.7)	

Values are the median and the 25^th^ and 75^th^ percentiles. Mann–Whitney U‐test or Kruskal-Wallis test was used for comparisons between the applicable and not applicable groups.

Abbreviations: Predicted FVC%, the FVC as a percentage of the predicted value.

**Table 6:** Relationship between the presence of obesity criteria outlier and pulmonary function in females aged ≥ 65 years.

		N	Predicted FVC%		P—value	FEV_1_/FVC ratio		P-value
			Median	(25%–75%)		Median	(25%–75%)	
**Waist circumference (cm)**	< 90 cm	207	118.4	(105.9–133.1)	0.003	79.8	(76.0–83.6)	0.727
	≥ 90 cm	20	109.3	(91.5–124.1)		79.1	(75.2–82.8)	
**Visceral fat area (cm**^**2**^**)**	< 100 cm^2^	176	108.8	(99.1–121.7)	0.002	79.6	(76.1–83.6)	0.685
	≥ 100 cm^2^	51	101.0	(89.1–115.6)		80.0	(75.5–83.5)	
**Waist-to-hip ratio**	< 0.85	144	108.9	(97.6–121.6)	0.183	79.6	(75.5–83.5)	0.345
	≥ 0.85	83	106.5	(96.6–117.3)		79.9	(76.8–84.0)	
**Body Mass Index**	< 25 kg/cm^2^	170	108.8	(99.0–120.9)	0.023	79.4	(75.9–83.2)	0.121
	≥ 25 kg/cm^2^	57	101.7	(91.7–115.9)		81.6	(77.2–84.3)	
**Sum of obesity criteria**	0	118	108.9	(99.0–121.3)	0.054	79.6	(75.8–83.6)	0.597
	1	50	107.6	(99.0–121.7)		79.4	(76.2–83.6)	
	2–4	59	101.7	(91.5–117.1)		80.3	(76.8–83.7)	

Values are the median and the 25^th^ and 75^th^ percentiles. Mann–Whitney U‐test or Kruskal-Wallis test was used for comparisons between the applicable and not applicable groups.

Abbreviations: Predicted FVC%, the FVC as a percentage of the predicted value.

### Relationship between the body composition index and pulmonary function

Table 7 shows the relationship between the body composition index and pulmonary function. In the younger male group, there was a significantly negative correlation between the predicted FVC% and body composition index (r = −0.102, p = 0.047 for body fat percentage; r = −0.108, p = 0.035 for body fat percentage of the trunk), as well as a significantly negative correlation between the FEV_1_/FVC ratio and body composition index (r = −0.111, p = 0.031 for the VFA; r = −0.136, p = 0.008 for WC). In the younger female group, there was a significantly positive correlation between the predicted FVC% and body composition index (r = 0.123, p = 0.004 for WC; r = 0.089, p = 0.034 for VFA; r = 0.091, p = 0.032 for BMI; r = 0.137, p = 0.01 for SMI), as well as a significantly negative correlation between the FEV_1_/FVC ratio and WC (r = −0.086, p = 0.041). In the older male group, there was a significantly positive correlation between the predicted FVC% and SMI (r = 0.210, p = 0.026). In the older female group, there was a significantly negative correlation between the predicted FVC% and body composition index (r = −0.139, p = 0.037 for body fat percentage; r = −0.138, p = 0.039 for body fat percentage of the trunk; r = −0.172, p = 0.010 for VFA).

**Table 7:** Relationships between body composition and pulmonary function.

		Male				Female			
		(n = 498)				(n = 789)			
		19–64 years		≥ 65 years		19–64 years		≥ 65 years	
		(n = 383)		(n = 115)		(n = 562)		(n = 227)	
		partial r_s_	P-value	partial r_s_	P-value	partial r_s_	P-value	partial r_s_	P-value
**Body fat percentage**	Predicted FVC%	-0.102	0.047	-0.135	0.155	-0.033	0.435	-0.139	0.037
	FEV_1_/FVC ratio	-0.066	0.197	-0.040	0.674	-0.024	0.577	0.034	0.612
**Body fat percentage of the trunk**	Predicted FVC%	-0.108	0.035	-0.145	0.127	-0.025	0.560	-0.138	0.039
	FEV_1_/FVC ratio	-0.053	0.303	-0.031	0.746	-0.028	0.514	0.037	0.580
**Waist circumference**	Predicted FVC%	-0.034	0.514	-0.120	0.206	0.123	0.004	-0.121	0.071
	FEV_1_/FVC ratio	-0.136	0.008	-0.128	0.180	-0.086	0.041	0.042	0.531
**Visceral fat area**	Predicted FVC%	-0.079	0.125	-0.087	0.364	0.089	0.034	-0.172	0.010
	FEV_1_/FVC ratio	-0.111	0.031	-0.031	0.744	-0.057	0.176	0.020	0.762
**Waist-to-hip ratio**	Predicted FVC%	-0.006	0.914	-0.065	0.496	0.072	0.089	-0.042	0.531
	FEV_1_/FVC ratio	-0.022	0.666	-0.024	0.800	-0.051	0.229	0.037	0.583
**Body Mass Index**	Predicted FVC%	-0.050	0.328	-0.109	0.253	0.091	0.032	-0.075	0.264
	FEV_1_/FVC ratio	-0.060	0.242	0.053	0.583	-0.054	0.203	0.015	0.819
**Skeletal Muscle Index**	Predicted FVC%	0.070	0.176	0.210	0.026	0.137	0.001	-0.020	0.765
	FEV_1_/FVC ratio	-0.029	0.578	0.116	0.223	-0.033	0.436	0.039	0.558

Spearman’s partial correlation analysis between body composition and pulmonary function was used.

This analysis was adjusted for smoking status, age (only for the FEV_1_/FVC ratio), and SMI (except when analyzing the SMI). Smoking status was categorized as current-smoker, ex-smoker, and non-smoker; these were included as dummy variables.

Abbreviations: Predicted FVC%, the FVC as a percentage of the predicted value.

### Relationships between HOMA-IR, IL-6, and the FEV_1_/FVC ratio in the younger male group

Tables 8 and 9 show the relationships between HOMA-IR, IL-6, and the FEV_1_/FVC ratio in the younger male group. There was no significant correlation between HOMA-IR, IL-6, and the FEV_1_/FVC ratio. Individuals with abnormal IL-6 levels had a significantly lower FEV_1_/FVC ratio than those with normal IL-6 levels (p = 0.045). However, there was no difference in the FEV_1_/FVC ratio between those with and without abnormal glucose tolerance.

**Table 8:** Relationships between pulmonary function, glucose tolerance, and inflammatory cytokine levels in males aged 19–64 years.

	N	FEV_1_/FVC ratio	
		partial r_s_	P-value
**IL-6**	371	-0.073	0.163
**HOMA-R**	371	0.031	0.557

Spearman’s partial correlation analysis was used to determine the relationship between body composition and pulmonary function.

This analysis was adjusted for confounders, including smoking status and age. Smoking status was categorized as current-smoker, ex-smoker, and non-smoker, which were included as dummy variables.

HOMA-IR, homeostasis model assessment of insulin resistance; IL-6, interleukin-6.

**Table 9:** Relationships between the FEV_1_/FVC ratio, glucose tolerance, and inflammatory cytokines in males aged 19–64 years.

		N	FEV_1_ / FVC ratio		P-value
			Median	(25%–75%)	
**IL-6**	≤ 4.0 pg/ml	360	81.8	(77.5–85.7)	0.045
	> 4.0 pg/ml	11	76.5	(73.8–82.5)	
**HOMA-IR**	< 2.5	361	81.7	(77.0–85.6)	0.207
	≥ 2.5	10	84.9	(79.1–87.4)	

Values are the median and the 25^th^ and 75^th^ percentiles. Mann–Whitney U‐test was used to compare the applicable and not applicable groups with respect to abnormal values of glucose tolerance and inflammatory cytokines.

HOMA-IR, homeostasis model assessment of insulin resistance; IL-6, interleukin-6.

### Relationship of the visceral fat area with HOMA-IR and IL-6 in the younger male group

Tables 10 and 11 show the relationship of VFA with HOMA-IR and IL-6 in the younger male group. VFA showed a significant positive correlation with HOMA-IR (r = 0.570, p< 0.001) and IL-6 (r = 0.254, p< 0.001). In addition, there were significantly more individuals with abnormal values for VFA than those without (p< 0.001 for both HOMA-IR and IL-6).

**Table 10:** Relationships among glucose tolerance, inflammatory cytokines, and visceral fat area in males aged 19–64 years.

	N	IL-6		HOMA-R	
		partial r_s_	P-value	partial r_s_	P-value
**Visceral fat area**	371	0.254	< 0.001	0.570	< 0.001

Spearman’s correlation analysis for glucose tolerance, inflammatory cytokines, and visceral fat area was used.

HOMA-IR, homeostasis model assessment of insulin resistance; IL-6, interleukin-6.

**Table 11:** Relationships between glucose tolerance, inflammatory cytokines, and visceral fat area.

		N	IL-6		P-value	HOMA-IR		P-value
			Median	(25%–75%)		Median	(25%–75%)	
**Visceral fat area**	< 100 cm^2^	196	0.8	(0.6–1.1)	< 0.001	0.6	(0.5–0.9)	< 0.001
	≥ 100 cm^2^	175	1.1	(0.7–1.6)		1.1	(0.8–1.5)	

Values are the median and the 25^th^ and 75^th^ percentiles. The p-value was obtained using the Mann–Whitney U‐test between the applicable and not applicable groups, in terms of abnormal visceral fat area.

HOMA-IR, homeostasis model assessment of insulin resistance; IL-6, interleukin-6.

## Discussion

This study examined the association between body composition and pulmonary function. In the younger age groups of both sexes, the FEV_1_/FVC ratio was significantly higher in the normal VFA group than in the obesity group (Table 3). However, after adjusting for confounding factors, the younger male group showed a significant negative correlation between the FEV_1_/FVC ratio and VFA only (Table 7). Because the FEV_1_/FVC ratio is used as a diagnostic criterion for obstructive pulmonary disease [23], VFA accumulation may increase the risk of obstructive pulmonary disease in young males. The relationship between the FEV_1_/FVC ratio and VFA has been reported in studies conducted in Korea [24] and the Netherlands [25], with none of the studies reporting a significant relationship. Further studies are needed to confirm these findings.

On the contrary, in the older female group, the predicted FVC% was significantly higher in the normal WC, VFA, and BMI groups than in the obesity group (Table 6). However, the older female group showed a significant negative correlation of the predicted FVC% with the body fat percentage, body fat percentage of the trunk, and VFA, after adjusting for confounding factors. The study results suggest that the increase of VFA may accelerate the decline of pulmonary function (predicted FVC%) in older females.

Metabolic syndrome affects the FEV_1_/FVC ratio through various factors such as increased glucose tolerance and inflammatory cytokines [8]. The present study showed no correlation among pulmonary function, glucose tolerance, and inflammatory cytokines. However, the FEV_1_/FVC ratio was significantly lower in young men with abnormal IL-6 levels than in those with normal IL-6 levels. In addition, there was a positive correlation between VFA and IL-6 concentration in the same group. Further, IL-6 concentration was significantly higher in individuals with abnormal VFA than in those without. Therefore, it is likely that IL-6 was positively correlated with VFA in younger men, which may have resulted in the decrease in the FEV_1_/FVC ratio. Previous studies compared visceral fat and IL-6 in the obstructive and non-obstructive pulmonary disease groups and showed that the former had significantly greater visceral fat and IL-6 levels than the latter [26, 27]. In addition, these studies compared VFA and IL-6 and analyzed the independent associations of obstructive pulmonary disease status and VFA on iL-6 plasma concentrations. The present findings are consistent with previous findings and suggest that excessive abdominal visceral fat contributes to increased IL-6 levels, which causes obstructive pulmonary disease in older adults [27].

In women, decreased estrogen and progesterone levels with menopause lead to increased visceral fat [28]. Furthermore, VFA and VFA/subcutaneous fat area after menopause are significantly higher than those before menopause [29]. In this study, only elderly women showed an association between VFA and predicted FVC%. The increase in visceral fat in older women in the menopausal group may have contributed to decreased respiratory compliance and consequently affected the association between VFA and predicted FVC%.

A study on Western participants reported negative correlations between WHR, BMI, and pulmonary function [30]. In this study, there was no relationship between WHR and pulmonary function; moreover, there was a relationship between BMI and pulmonary function in females only. This result was influenced by the physical characteristics of the Japanese population. The percentage of male and female Americans with BMI ≥ 30 kg/m^2^ is 35.0% and 40.4%, respectively [31], while the corresponding values for Japanese are 4.5% and 3.9% [32]. The corresponding values in the present study were 3.8% and 3.0%. It has been shown that FVC and FEV_1_ significantly decrease when the BMI and WHR are ≥ 30 and ≥ 1.01, respectively [33]. Further, it has been suggested that the effect on pulmonary function remains small except in cases with extreme obesity [34]. Since the participants in this study had less extreme obesity, it could have attributed to the findings of no relationships of BMI and WHR with pulmonary function.

This study showed a strong association between WC, VFA, and pulmonary function. It has been reported that the VFA to subcutaneous fat ratio is higher in the Japanese than in other races [35, 36]. Therefore, the Japanese may be more affected by diseases related to visceral fat than other races. It has been suggested that VFA measurement is important as a disease marker for pulmonary disease in the Japanese. The effects of obesity on pulmonary function are more associated with FVC than with FEV_1_ [37]. Furthermore, VFA is positively correlated with intra-abdominal visceral fat volume [38], with numerous studies examining the relationship between FVC and FEV_1_ (VFA, FVC, and FEV_1_ showed negative correlations) [25, 39–41]. However, there have been few reports on the relationship between VFA and the FEV_1_/FVC ratio. In this study, VFA in young males was related to the FEV_1_/FVC ratio but did not predict FVC%.

The present study examined the relationship between body composition and pulmonary function in the general population of the Aomori Prefecture lacking pulmonary diseases. There was a weak but significant association between VFA and the FEV_1_/FVC ratio in the younger male group. The possibility that obesity prevention may improve pulmonary function from such a period of no apparent disease is immensely important from an epidemiological perspective.

There were some limitations to the present study. First, because this was a cross-sectional study, we could not prove causality. Longitudinal studies are needed to reveal the causal relationship between body composition and pulmonary function. Second, all the participants were volunteers; therefore, they might have been more interested in their health status and could have been healthier than the general population. Nevertheless, even with these limitations, the present study measured the VFA in a sample size relatively larger than that of previous studies. Our study provided basic evidence regarding the relationships between body composition and pulmonary function.

## Conclusions

This study, which examined the association between body composition and pulmonary function, provides basic data for the prevention and improvement of obstructive pulmonary diseases. The FEV_1_/FVC ratio was significantly higher in the younger male group with normal VFA than in those with obesity. Moreover, there was a significantly negative correlation of the FEV_1_/FVC ratio with WC and VFA after adjusting for confounding factors in the same group. These results suggest that increased VFA could promote the development of obstructive pulmonary disease. By contrast, in the older female group, the predicted FVC% was significantly higher in the normal WC, VFA, and BMI groups than in the obesity group. In addition, there was a significantly negative correlation of the predicted FVC% with body fat percentage, body fat percentage of the trunk, and VFA. The study results suggest that increased VFA may accelerate the decline of pulmonary function (predicted FVC%) in the older female group. In addition, IL-6 may be related to a decrease in the FEV_1_/FVC ratio associated with visceral fat accumulation. Furthermore, VFA measurement may be an important disease marker for pulmonary disease among the Japanese population.

## References

[1] Burrows B, Knudson RJ, Cline MG, Lebowitz MD. Quantitative relationships between cigarette smoking and ventilatory function. Am Rev Respir Dis. 1976;155: 195–205. 10.1164/arrd.1977.115.2.195 842934

[2] Janssens JP, Pache JC, Nicod LP. Physiological changes in respiratory function associated with ageing. Eur Respir J. 1999;13: 197–205. 10.1034/j.1399-3003.1999.13a36.x 10836348

[3] Park CH, Yi Y, Do JG, Lee YT, Yoon KJ. Relationship between skeletal muscle mass and lung function in Korean adults without clinically apparent lung disease. Medicine (Baltimore). 2018;97: e12281 10.1097/MD.0000000000012281 30212965 PMC6155967

[4] Salome CM, King GG, Berend N. Physiology of obesity and effects on lung function. J Appl Physiol. 2010;108: 206–211. 10.1152/japplphysiol.00694.2009 19875713

[5] Donaldson GC, Seemungal TA, Bhowmik A, Wedzicha JA. Relationship between exacerbation frequency and lung function decline in chronic obstructive pulmonary disease. Thorax. 2002;57: 847–852. 10.1136/thorax.57.10.847 12324669 PMC1746193

[6] DeLorey DS, Wyrick BL, Babb TG. Mild-to-moderate obesity: implications for respiratory mechanics at rest and during exercise in young men. Int J Obes. 2005;29: 1039–1047. 10.1038/sj.ijo.0803003 15917840

[7] Poulain M, Doucet M, Major GC, Drapeau V, Sériès F, Boulet LP, et al The effect of obesity on chronic respiratory diseases: pathophysiology and therapeutic strategies. CMAJ. 2006;174: 1293–1299. 10.1503/cmaj.051299 16636330 PMC1435949

[8] Baffi CW, Wood L, Winnica D, Strollo PJ Jr, Gladwin MT, Que LG, et al Metabolic syndrome and the lung. Chest. 2016;149: 1525–1534. 10.1016/j.chest.2015.12.034 26836925 PMC4944780

[9] Enzi G, Gasparo M, Biondetti PR, Fiore D, Semisa M, Zurlo F. Subcutaneous and visceral fat distribution according to sex, age, and overweight, evaluated by computed tomography. Am J Clin Nutr. 1986;44: 739–746. 10.1093/ajcn/44.6.739 3788827

[10] Kooy K, Leenen R, Seidell JC, Deurenberg P, Visser M. Abdominal diameters as indicators of visceral fat: comparison between magnetic resonance imaging and anthropometry. Br J Nutr. 1993;70: 47–58. 10.1079/bjn19930104 8399118

[11] Jensen MD, Kanaley JA, Reed JE, Sheedy PF. Measurement of abdominal and visceral fat with computed tomography and dual-energy x-ray absorptiometry. Am J Clin Nutr. 1995;61: 274–278. 10.1093/ajcn/61.2.274 7840063

[12] Naka T, Han I, Keii T, Kasahara Y, Nishizawa M, Miyoshi T, et al Body composition analysis using segmental bioelectrical impedance in healthy participants. Jikeikai Med J. 2005;120: 35–44.

[13] Ryo M, Maeda K, Onda T, Katashima M, Okumiya A, Nishida M, et al A new simple method for the measurement of visceral fat accumulation by bioelectrical impedance. Diabetes Care. 2005;28: 451–453. 10.2337/diacare.28.2.451 15677816

[14] Ida M, Hirata M, Odori S, Mori E, Kondo E, Fujikura J, et al Early changes of abdominal adiposity detected with weekly dual bioelectrical impedance analysis during calorie restriction. Obesity (Silver Spring). 2013;21: 350–353. 10.1002/oby.20300 23703886

[15] Kadokawa T, Haneda M, Tominaga M, Yamada N, Iwamoto Y, Tajima N, et al Report of the Japan Diabetes Society's Committee on the diagnostic criteria for diabetes mellitus and glucose metabolism disorder. J Jpn Diabetes Soc. 2008;51: 281–283.

[16] The Examination Committee of Criteria for ‘Obesity Disease’ in Japan, Japan Society for the Study of Obesity, New Criteria for ‘Obesity Disease’ in Japan. Circ J. 2002;66: 987–992. 10.1253/circj.66.987 12419927

[17] Baumgartner RN, Koehler KM, Gallagher D, Romero L, Heymsfield SB, Ross RR, et al Epidemiology of sarcopenia among the elderly in New Mexico. Am J Epidemiol. 1998;147: 755–763. 10.1093/oxfordjournals.aje.a009520 9554417

[18] World Health Organization. Definition, diagnosis and classification of diabetes mellitus and its complications: report of a WHO consultation Part 1, Diagnosis and classification of diabetes mellitus. World Health Organization 1999.

[19] Choi HS, Choi CW, Park MJ, Kang HM, Yoo JH. Clinical value of a desktop spirometer (HI-801) for spirometry screening. Tuberc Respir Dis. 2007;62(4): 276–283.

[20] Tojo N, Suga H, Kambe M. Lung function testing—the Official Guideline of the Japanese Respiratory Society. Rinsho Byori. 2005;53(1): 77–81. 15724494

[21] Kubota M, Kobayashi H, Quanjer PH, Omori H, Tatsumi K, Kanazawa M. Reference values for spirometry, including vital capacity, in Japanese adults calculated with the LMS method and compared with previous values. Respir Investig. 2014;52(4): 242–250. 10.1016/j.resinv.2014.03.003 24998371

[22] Hara K, Horikoshi M, Yamauchi T, Yago H, Miyazaki O, Ebinuma H, et al Measurement of the high–molecular weight form of adiponectin in plasma is useful for the prediction of insulin resistance and metabolic syndrome. Diabetes Care. 2006;29: 1357–1362. 10.2337/dc05-1801 16732021

[23] Griner PF, Mayewski RJ, Mushlin AI, Greenland P. Selection and interpretation of diagnostic tests and procedures. Ann Intern Med. 1981;94: 557–592. 6452080

[24] Choe EK, Kang HY, Lee Y, Choi SH, Kim HJ, Kim JS. The longitudinal association between changes in lung function and changes in abdominal visceral obesity in Korean non-smokers. PLoS ONE. 2018;13: e0193516 10.1371/journal.pone.0193516 29474424 PMC5825142

[25] Thijs W, Dehnavi RA, Hiemstra PS, de Roos A, Melissant CF, Janssen K, et al Association of lung function measurements and visceral fat in men with metabolic syndrome. Respir Med. 2014;108: 351–357. 10.1016/j.rmed.2013.10.003 24239315

[26] van den Borst B, Gosker H, Koster A, Kritchevsky S, Liu Y, Meibohm B, et al Obstructive lung disease is associated with increased abdominal visceral fat and elevated systemic adipocytokines. Eur Respir J. 2011; 38: 1887.

[27] van den Borst B, Gosker HR, Koster A, Yu B, Kritchevsky SB, Liu Y, et al The influence of abdominal visceral fat on inflammatory pathways and mortality risk in obstructive lung disease. Am J Clin Nutr. 2012;96: 516–526. 10.3945/ajcn.112.040774 22811442 PMC3417214

[28] Zamboni M, Armellini F, Milani MP, De Marchi M, Todesco T, Robbi R, et al Body fat distribution in pre and post-menopausal women: metabolic and anthropometric variables and their inter-relationships. Int J Obes. 1992;16: 495–504. 1323546

[29] Kotani K, Tokunaga K, Fujioka S, Kobatake T, Keno Y, Yoshida S, et al Sexual dimorphism of age-related changes in whole-body fat distribution in the obese. Int J Obes. 1994;18: 207–212 8044194

[30] Leone N, Courbon D, Thomas F, Bean K, Jégo B, Leynaert B, et al Lung function impairment and metabolic syndrome: the critical role of abdominal obesity. Am J Respir Crit Care Med. 2009;179: 509–516. 10.1164/rccm.200807-1195OC 19136371

[31] Flegal KM, Kruszon-Moran D, Carroll MD, Fryar CD, Ogden CL. Trends in obesity among adults in the United States, 2005 to 2014. JAMA. 2016;315: 2284–2291. 10.1001/jama.2016.6458 27272580 PMC11197437

[32] Ministry of Health, Labour and Welfare. Official reports of the National Health and Nutrition Survey 2017. https://www.mhlw.go.jp/content/000451755.pdf. Accessed March 29, 2020.

[33] Wannamethee SG, Shaper AG, Whincup PH. Body fat distribution, body composition, and respiratory function in elderly men. Am J Clin Nutr. 2005;82(5): 996–1003. 10.1093/ajcn/82.5.996 16280430

[34] Mohamed EI, Maiolo C, Iacopino L, Pepe M, Daniele N, Lorenzo A. The impact of body-weight components on forced spirometry in health Italians. Lung. 2002;180(3): 149–159. 10.1007/s004080000089 12177729

[35] Tanaka S, Horimai C, Katsukawa F. Ethnic differences in abdominal visceral fat accumulation between Japanese, African-Americans, and Caucasians: a meta-analysis. Acta Diabetol. 2003;40: 302–304. 10.1007/s00592-003-0093-z 14618500

[36] Kadowaki T, Sekikawa A, Murata K, Maegawa H, Takamiya T, Okamura T, et al Japanese men have larger areas of visceral adipose tissue than Caucasian men in the same levels of waist circumference in a population-based study. Int J Obes. 2006;30(7): 1163–1165. 10.1038/sj.ijo.0803248 16446744

[37] Rowe A, Hernandez P, Kuhle S, Kirkland S. The association between anthropometric measures and lung function in a population-based study of Canadian adults. Respir Med. 2017;131: 199–204. 10.1016/j.rmed.2017.08.030 28947030

[38] Ryo M, Kishida K, Nakamura T, Yoshizumi T, Funahashi T, Shimomura I. Clinical significance of visceral adiposity assessed by computed tomography: a Japanese perspective. World J Radiol. 2014;6: 409–416. 10.4329/wjr.v6.i7.409 25071881 PMC4109092

[39] Park YS, Kwon HT, Hwang SS, Choi SH, Cho YM, Lee J, et al Impact of visceral adiposity measured by abdominal computed tomography on pulmonary function. J Korean Med Sci. 2011;26: 771–777. 10.3346/jkms.2011.26.6.771 21655063 PMC3102871

[40] de Oliveira PD, Wehrmeister FC, Horta BL, Pérez-Padilla R, de França GV, Gigante DP, et al Visceral and subcutaneous abdominal adiposity and pulmonary function in 30-year-old adults: a cross-sectional analysis nested in a birth cohort. BMC Pulm Med. 2017;17: 157 10.1186/s12890-017-0510-7 29179743 PMC5704528

[41] Inomoto A, Fukuda R, Deguchi J, Kato G, Kanzaki R, Hiroshige K, et al The association between the body composition and lifestyle affecting pulmonary function in Japanese workers. J Phys Ther Sci. 2016;28: 2883–2889. 10.1589/jpts.28.2883 27821955 PMC5088146

